# Bio-inspired carbon-based artificial muscle with precise and continuous morphing capabilities

**DOI:** 10.1093/nsr/nwae400

**Published:** 2024-11-08

**Authors:** Xiaodong Li, Meiping Li, Mingjia Zhang, Qin Liu, Deyi Zhang, Wenjing Liu, Xingru Yan, Changshui Huang

**Affiliations:** CAS Key Laboratory of Organic Solids, Beijing National Laboratory for Molecular Sciences (BNLMS), CAS Research/Education Center for Excellence in Molecular Sciences, Institute of Chemistry, Chinese Academy of Sciences, Beijing 100190, China; CAS Key Laboratory of Organic Solids, Beijing National Laboratory for Molecular Sciences (BNLMS), CAS Research/Education Center for Excellence in Molecular Sciences, Institute of Chemistry, Chinese Academy of Sciences, Beijing 100190, China; College of Physics and Optoelectronic Engineering, Ocean University of China, Qingdao 266100, China; Key Laboratory of Theoretical Organic Chemistry and Functional Molecules, Ministry of Education, School of Chemistry and Chemical Engineering, Hunan University of Science and Technology, Xiangtan 411201, China; CAS Key Laboratory of Organic Solids, Beijing National Laboratory for Molecular Sciences (BNLMS), CAS Research/Education Center for Excellence in Molecular Sciences, Institute of Chemistry, Chinese Academy of Sciences, Beijing 100190, China; School of Chemical Sciences, University of Chinese Academy of Sciences, Beijing 100049, China; CAS Key Laboratory of Organic Solids, Beijing National Laboratory for Molecular Sciences (BNLMS), CAS Research/Education Center for Excellence in Molecular Sciences, Institute of Chemistry, Chinese Academy of Sciences, Beijing 100190, China; School of Chemical Sciences, University of Chinese Academy of Sciences, Beijing 100049, China; CAS Key Laboratory of Organic Solids, Beijing National Laboratory for Molecular Sciences (BNLMS), CAS Research/Education Center for Excellence in Molecular Sciences, Institute of Chemistry, Chinese Academy of Sciences, Beijing 100190, China; School of Chemical Sciences, University of Chinese Academy of Sciences, Beijing 100049, China; CAS Key Laboratory of Organic Solids, Beijing National Laboratory for Molecular Sciences (BNLMS), CAS Research/Education Center for Excellence in Molecular Sciences, Institute of Chemistry, Chinese Academy of Sciences, Beijing 100190, China; School of Chemical Sciences, University of Chinese Academy of Sciences, Beijing 100049, China

**Keywords:** graphdiyne, artificial muscle, carbon-based materials, stimulus-responsive materials, actuator

## Abstract

In the face of advancements in microrobotics, intelligent control and precision medicine, artificial muscle actuation systems must meet demands for precise control, high stability, environmental adaptability and high integration miniaturization. Carbon materials, being lightweight, strong and highly conductive and flexible, show great potential for artificial muscles. Inspired by the butterfly's proboscis, we have developed a carbon-based artificial muscle, hydrogen-substituted graphdiyne muscle (HsGDY-M), fabricated efficiently using an emerging hydrogen-substituted graphdiyne (HsGDY) film with an asymmetrical surface structure. This muscle features reversible, rapid and continuously adjustable deformation capabilities similar to the butterfly's proboscis, triggered by the conversion of carbon bonds. The size of the HsGDY-M can be tuned by changing the HsGDY film width from ∼1 cm to 100 μm. Our research demonstrates HsGDY-M's stability and adaptability, maintaining performance at temperatures as low as −25°C. This artificial muscle was successfully integrated into a robotic mechanical arm, allowing it to swiftly adjust its posture and lift objects up to 11 times its own weight. Its beneficial responsiveness is transferable, enabling the transformation of ‘inert’ objects like copper foil into actuators via surface bonding. Because of its super sensitive and rapid deformation, HsGDY-M was applied to create a real-time tracking system for human finger bending movements, achieving real-time simulation and large-hand-to-small-hand control. Our study indicates that HsGDY-M holds significant promise for advancing smart robotics and precision medicine.

## Teaser

Inspired by a butterfly's proboscis, this carbon-based artificial muscle offers precision, rapid morphing and adaptability, heralding potential breakthroughs in robotics and enabling real-time tracking for medical applications.

## INTRODUCTION

With the advancement of current technology, artificial muscles, as a type of biomimetic material, have become a key technology in the fields of intelligent robotics and healthcare [1]. Their significance lies in their ability to mimic the functions of natural muscles, providing power and support, while also possessing characteristics such as self-repair, high elasticity and fast response times, which are unparalleled by traditional mechanical actuators [2,3]. As the population ages and the demand in the labor market changes, artificial muscle technology is proving to be invaluable in assistive devices, wearable technology and a wide range of medical applications [4–6].

Artificial muscles come in various types, and new materials and manufacturing technologies offer multiple options for preparing artificial muscles that convert electrical, chemical, thermal or light energy into mechanical energy. Specifically, these materials include nanocomposites [7], shape memory alloys [8,9], dielectric elastomers [10,11], ion polymer-metal composites [12], conductive polymers [13,14] and biomass fibers [15–17]. Each material has its unique characteristics; for example, electroactive polymers can generate significant force and displacement under an electric field, while shape memory alloys can return to their preset shape when triggered by a certain temperature [18,19]. However, these materials often face challenges such as durability, environmental adaptability and slow response times. Future developments in artificial muscles demand higher standards, including improved material stability, environmental adaptability and precision in rapid operations. In this field, carbon materials exhibit unique advantages due to their excellent mechanical properties, electrochemical characteristics, high durability and tunable microstructures [20–25]. Additionally, the miniaturization of artificial muscles is an important trend, especially in micro-medical devices and microrobots, where precise control and application can be achieved [26]. Addressing these challenges hinges on the development of innovative artificial muscle materials.

Inspired by the proboscis of a butterfly, we have developed a new carbon-based artificial muscle material, hydrogen-substituted graphdiyne muscle (HsGDY-M). HsGDY-M features an asymmetric hierarchical porous structure, which not only retains the traditional advantages of carbon materials, such as excellent stability and environmental adaptability, allowing it to operate at −25°C, but also possesses the ability for fast, continuous and precisely controllable deformation. When integrated into a robot's mechanical arm, HsGDY-M enables rapid posture adjustment and can lift objects up to 11 times its own weight. Additionally, its beneficial response capabilities are transferable, allowing ‘inert’ objects to be converted into actuators through surface adhesion. To demonstrate its potential for miniaturization, HsGDY-M was used to create a real-time tracking system for human finger movements, capable of simulating the control of a large hand over a small hand in real time. This study indicates that HsGDY-M holds significant promise in advancing intelligent robotics and precision medicine.

## RESULTS AND DISCUSSION

**Figure 1.**
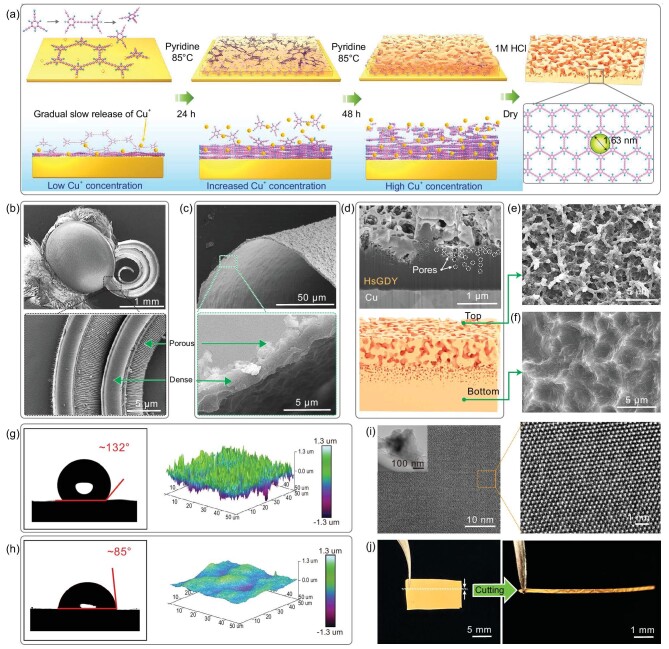
(a) Schematic diagram of the HsGDY-M synthesis process, including skeleton structure and pore size (1.63 nm). (b) Scanning electron microscopy (SEM) image of a butterfly's head, highlighting the asymmetrical structure of its proboscis, with a dense side and a porous side. (c) SEM images of HsGDY-M. (d) Cross-sectional image of HsGDY-M prepared using a focused ion beam on a copper foil substrate, and its schematic diagram. (e) SEM image of top surface. (f) SEM image of bottom surface. (g) Top surface morphology of HsGDY-M using atomic force microscopy (AFM) and contact angles (∼132°) with water. (h) Bottom surface morphology of HsGDY-M using AFM and contact angles (∼85°) with water. (i) High-resolution transmission electron microscopy image of HsGDY-M, with an inset of an SEM image. (j) The electronic image of independent HsGDY-M film, with its cut shape on the right.

Inspired by the butterfly proboscis's unique structure and functional characteristics, we synthesized HsGDY-M through a one-step mild liquid-phase reaction, as illustrated in Fig. 1a. Specifically, HsGDY-M was synthesized via a carbon–carbon coupling reaction of 1,3,5-triethynylbenzene, resulting in a material with apertures ∼1.63 nm in size on a 2D plane. During the growth process, the copper foil continuously released copper ions under the influence of pyridine, which served as a catalyst for the coupling reaction. Initially, the film grew slowly and was compact due to the limited release of copper ions. As time progressed, the concentration of copper ions gradually increased, accelerating the reaction and leading to a looser and more porous film structure. After removing the HsGDY film from the copper foil with 1 M hydrochloric acid (HCl), the film was dried to yield an independently supported HsGDY-M. Scanning electron microscope (SEM) images, as depicted in Figs 1b and S2, reveal that the butterfly's proboscis exhibits a distinctively curved and asymmetric structure. This dense and porous asymmetry combination provides the proboscis with structural stability and a capacity for deformation. Similarly, the HsGDY-M we prepared displays an asymmetric porous structure, as seen in the SEM images in Fig. 1c and Fig. S4. This feature aligns with the asymmetric structure of the butterfly proboscis (Fig. 1b). Here, the side of HsGDY-M facing away from the copper foil is defined as the top surface, while the side in contact with the copper foil forms the bottom surface. The SEM images of the HsGDY-M cross-section in Fig. 1d and Fig. S5 illustrate a transition in the pore structure from porous at the top to dense at the bottom, creating an asymmetric gradient throughout the film. Figure 1e and f clearly demonstrate significant differences between the top and bottom surfaces; the top surface is rough and porous, whereas the bottom surface is relatively smooth and dense. Moreover, as shown in Fig. 1g and h, the top and bottom surfaces differ in their contact angles with water and in surface roughness, as characterized by atomic force microscopy (AFM). Both surfaces also exhibit hydrophilicity towards acetone, enabling the liquid to spread across the surfaces within ∼20 milliseconds, as illustrated in Fig. S6. The consistency in chemical structure between the top and bottom surfaces was confirmed through Raman spectroscopy (Fig. S7) and X-ray photoelectron spectroscopy (XPS) (Fig. S8a). High-resolution transmission electron microscopy (HRTEM) in Fig. 1i displays the clear lattice of HsGDY, indicating its excellent crystalline characteristics. The HsGDY-M can be directly tailored into various shapes according to specific requirements and Fig. 1j shows a photo of the film cut into long strips.

**Figure 2.**
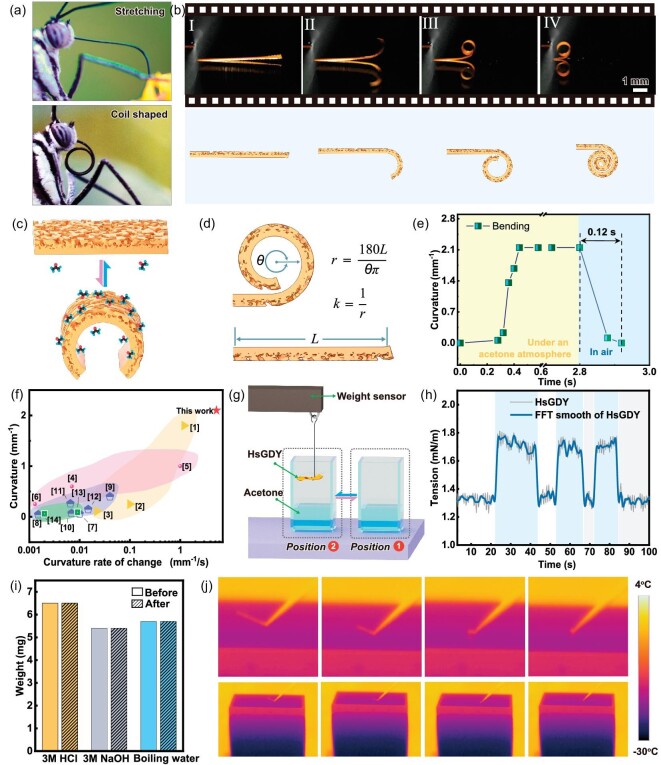
(a) The photos show the proboscis of butterflies, demonstrating their ability to coil and stretch flexibly (Image source: Pixabay, free-to-use license). (b) Electronic image of HsGDY-M mimicking the coiling and stretching of a butterfly's proboscis, accompanied by its corresponding schematic diagram. (c) Schematic showing deformation of HsGDY-M under the influence of acetone atmosphere. (d) Schematic illustration of the definition of curvature. (e) The plot of curvature against time for the HsGDY-M. (f) Comparison of the curvature and curvature change rate of the HsGDY with those of typical single-layer responsive materials in the literature. (g) Experimental set-up diagram of the weight change of HsGDY adsorbed acetone. (h) Changes in weight of HsGDY before and after adsorption of acetone molecules over time. (i and j) Infrared photos of the response of HsGDY-M to acetone molecules at −25°C.

Figure 2a shows the proboscis of butterflies in various states, emphasizing the transition from a coiled state to an extended state used for consuming nectar. At rest, the proboscis curls tightly under the head, minimizing space. Conversely, it can extend to match the butterfly's body length during feeding, facilitating nectar extraction. After successfully simulating the structure of a butterfly's proboscis, we investigated the stimulus-response performance of HsGDY-M. It demonstrated reactivity to acetone vapor with rapid morphological transitions from stretching to curling, as shown in Fig. 2b and Video 1. Upon removal of the acetone vapor, it reverts to its original state, effectively mimicking the dynamic behavior of a butterfly's proboscis. We conducted a quantitative analysis of the rapid deformation characteristics of HsGDY-M under acetone stimulation, as illustrated in Fig. 2c. Initially, the curvature deformation was defined, as shown in Fig. 2d. By analyzing its deformation curvature and time, we observed that HsGDY-M transitions from a stretched to a fully curled state within 0.25 seconds, achieving a curvature of 2.1 mm^−1^. Conversely, it reverts to a stretched state from a fully curled state in just 0.12 seconds, as shown in Fig. 2e. The rapid nature of this transition can be attributed to the inherent rigidity of HsGDY-M. During the curling process, elastic potential energy is accumulated, which is then utilized to facilitate a quick return to the stretched state. From the perspective of length variation, it demonstrates an excellent response speed—moving from 10 mm to 1 mm within 0.15 seconds, with a linear velocity of up to 60 mm/s. HsGDY-M, composed of a single material, can prevent interface stress concentration during deformation. Figure 2f presents an Ashby plot comparing the rate of curvature change and curvature among various single-layer, stimulus-responsive materials. HsGDY-M exhibits superior performance compared to other single-layer materials, including polymers [27], hydrogels [28], MXenes [29,30] and various carbon-based materials [25,31]. For detailed comparative data, refer to Table S1. Furthermore, we employed a precise *in-situ* weighing method to determine the adsorption rate of acetone molecules on HsGDY-M, as depicted in Fig. 2g and h. Upon altering the atmosphere to introduce acetone vapor, HsGDY-M reached adsorption saturation in just 1.5 seconds, evidenced by a stable mass reading. This rapid saturation demonstrates the high response speed of HsGDY in dynamic environments, underscoring its efficiency when interacting with acetone molecules. We assessed the stability of HsGDY-M under acidic, alkaline and hot water conditions. After soaking in 3 M hydrochloric acid, 3 M sodium hydroxide solutions and boiling water for 2 hours, the mass of HsGDY-M remained nearly unchanged, as shown in Fig. 2i, demonstrating its excellent stability. This is further supported by the XPS results shown in Fig. S8b. Low temperatures often present a challenging condition that can severely impair the performance of stimulus-responsive materials, sometimes leading to failure. However, HsGDY-M retains its response characteristics even at temperatures as low as −25°C, as evidenced by the infrared camera images in Fig. 2j. Furthermore, the decrease in temperature has a minimal impact, only slightly reducing the response speed, as evidenced in Fig. S10c. This capability significantly broadens the potential applications of HsGDY-M, making it suitable for environments with prevalent cold conditions.

**Figure 3.**
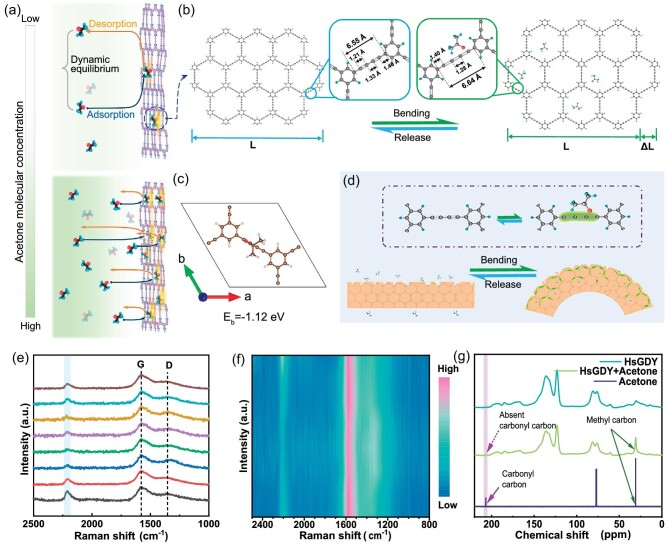
(a) Illustration of the adsorption dynamic equilibrium of acetone molecules on HsGDY. (b) The interaction between acetone molecules and HsGDY, especially how they induce changes in bond length, leading to changes in molecular skeleton length. (c) The configuration with the highest binding energy for acetone adsorption on HsGDY. (d) Schematic deformation diagram caused by asymmetric acetone adsorption on HsGDY-M. (e) *In-situ* Raman characterization of HsGDY before and after adsorption of acetone molecules. (f) Contour plot of *in-situ* Raman spectroscopy. (g) Solid NMR spectrum changes before and after acetone adsorption.

The shape change of HsGDY-M in response to varying acetone vapor concentrations is primarily a result of the adsorption dynamics of acetone molecules on its surface. Benefiting from the hierarchical porous structure of HsGDY-M, the adsorption and desorption processes quickly reach a stable dynamic equilibrium, as illustrated in Fig. 3a. An increase in concentration leads to more acetone molecules binding to HsGDY-M. The equilibrium is sensitive to changes in acetone concentration, enabling controlled deformation by adjusting the vapor concentration. Therefore, we have developed a simple and reliable method for the controllable adjustment of HsGDY-M shape variables by leveraging a natural acetone vapor concentration gradient that forms above the liquid surface. By altering the spatial height of HsGDY-M within this gradient, we can induce rapid and adaptive deformations corresponding to specific vapor concentrations, thus achieving controllable, continuous shape changes. HsGDY-M exhibits varying deformation states at different heights above the liquid surface, with a smaller deformation observed at greater heights, as detailed in Fig. S9c and d. When the height is fixed, HsGDY-M maintains a stable deformation state, which no longer changes with time and only depends on the distance from the liquid surface (Figs S10 and S11). Therefore, the naturally formed vapor concentration gradient provides a convenient and rapid way to control the size of deformation. Crucially, Fig. S12 demonstrates that HsGDY-M adapts continuously and rapidly to changes in vapor concentration throughout the deformation process, offering a significant advantage over systems that only respond in binary (on/off) states. At the molecular level, the adsorption of acetone onto the HsGDY framework triggers a charge transfer [32]. Specifically, a portion of the electron density shifts from the acetylene bonds in the framework to the adsorbed acetone. This redistribution of charge leads to the conversion of acetylene bonds into ethylene bonds, which leads to longer bond lengths (Fig. 3b). Acetone acts as a molecular switch, playing an indispensable role in the reversible transformation of carbon–carbon triple bonds into double bonds within the HsGDY structure. Theoretical calculations facilitate the identification of the most probable binding sites for acetone molecules on the HsGDY framework, as shown in Fig. 3c. Specifically, they indicate that acetone molecules adsorbed above the acetylene bonds possess a binding energy of −1.12 eV, representing the most stable structural configuration. The asymmetric morphological structure of the HsGDY-M acts as a bridge between microscale chemical bond changes and macroscale shape deformation (Fig. 3d). The top surface's loose and porous structure facilitates more comprehensive acetone adsorption, leading to a higher density of bond conversion and causing the HsGDY-M to bend toward the bottom surface. *In-situ* Raman spectroscopy provides further insights into the conversion mechanism from acetylene to ethylene, revealing different vibrational displacements during the adsorption process of acetone molecules. As shown in Fig. 3e, the acetylene bond peak near 2200 cm^−1^ in HsGDY notably weakens upon acetone adsorption, while the D peak becomes more distinct. Notably, these Raman spectral changes revert to their original state once the acetone atmosphere is removed, indicating a fully reversible process, as shown in Fig. 3f. The acetylene bond peak in HsGDY's Raman spectra does not disappear entirely after acetone adsorption. This is attributed to the fact that, under standard conditions (25°C and atmospheric pressure), acetone molecules cannot fully adsorb and occupy all acetylene bond sites, resulting in a reduction in the intensity of the acetylene bonds. *In-situ* infrared spectroscopy also shows significant adsorption of acetone molecules on HsGDY (Fig. S15). Further validation of this interaction is achieved through solid-state nuclear magnetic resonance (NMR), as shown in Fig. 3g. Comparative analysis of HsGDY's solid-state NMR spectra before and after acetone adsorption yields noteworthy results. The methyl carbon peak in HsGDY significantly strengthens after acetone adsorption, while the carbonyl carbon peak characteristic of acetone molecules is no longer observable. These changes in the NMR spectrum indirectly indicate a strong interaction between the acetylene bonds in the HsGDY framework and the carbonyl of the acetone molecules [32,33]. This solid-state NMR evidence supports the previously discussed findings and provides a deeper molecular-level understanding of how acetone molecules affect the HsGDY structure.

**Figure 4.**
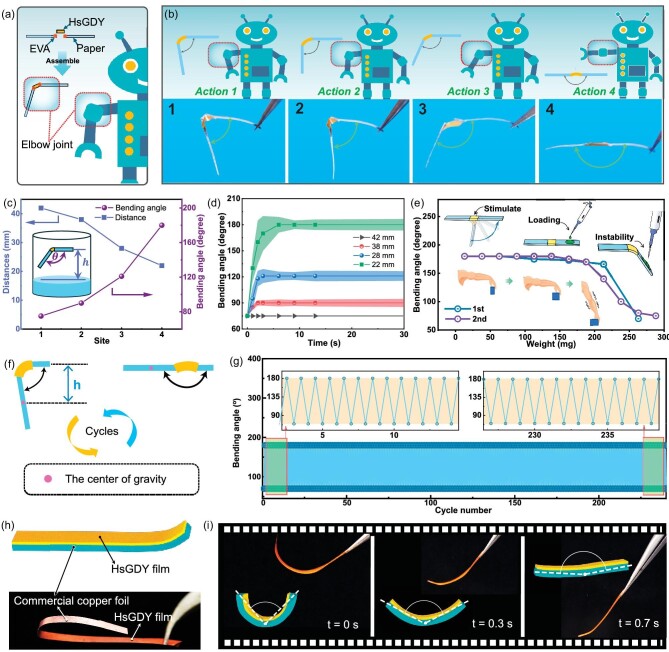
(a) Schematic diagram of the assembly of HsGDY into a robot elbow joint. (b) Photos of the controllable motion arm assembled by HsGDY-M, which can be used as an arm application for robots, and a schematic diagram of the bending angle of the HsGDY-M assembled arm. (c) The liquid level height at which the robotic arm is placed in different spatial positions and the angle at which the robotic arm bends under corresponding conditions. (d) The variation of the bending angle of the robotic arm over time at different heights. (e) Maximum drive weight tests were conducted on HsGDY-M, evaluating its load-bearing capacity. (f and g) Cycling stability test driven by HsGDY-M. (h) Schematic diagram and electronic photo of HsGDY covering single-sided copper foil. (i) Electronic photos of the deformation process of copper foil directly driven by HsGDY.

Robotic arms require materials that support continuous, adaptable movements beyond basic lifting and lowering. These materials must enable precise, rapid and easily controllable deformations to optimize utility. Additionally, their control methods should be simple and cost effective. However, many existing materials only offer limited state changes or are hampered by slow response times, which do not meet the necessary efficiency standards. In contrast, HsGDY-M material demonstrates significant potential for rapid, controllable and continuous deformation, offering a practical solution to these challenges. As shown in Fig. 4a, HsGDY-M serves as the active component in an artificial muscle for a mechanical arm model. This model is assembled by attaching paper tape arms to both ends of the HsGDY-M using a small amount of ethylenevinyl acetate copolymer (EVA), with these paper arms providing initial loads through specified masses. This set-up facilitates an in-depth analysis of the HsGDY-M's deformation response under various stress levels. Figure 4b illustrates the HsGDY-M robotic arm in action, demonstrating its capability to perform both lifting and lowering motions. The combination of the top diagram and motion photographs below clearly showcases the arm's ability to pause at any chosen angle, offering a level of control not typically achievable with other gas-stimulus-responsive materials. Further details of this process are given in Video 2. We have developed a straightforward yet effective method to control the posture of a robotic arm by adjusting its bending angle. As demonstrated in Fig. 4c, this is achieved by varying the arm's height above the liquid level. The greater the height, the smaller the bending angle. Importantly, as depicted in Fig. 4d, once a specific height is set, the angle of the arm remains constant over time, illustrating the material’s precise and consistent response to changes in position. To evaluate the load-bearing capacity of a robotic arm constructed from HsGDY-M, we measured its maximum capacity in a horizontal orientation. The arm was precisely positioned at a height of 22 mm above the liquid surface, ensuring a horizontal alignment as depicted in Fig. 4e, which replicates typical operational conditions. During the test, water was incrementally added to one end of the arm using a pipette, and the changes in the arm's angle were recorded twice throughout this procedure. The results demonstrate that the robotic arm can support a weight of 180 mg, which is 11 times the weight of the HsGDY material itself. As demonstrated in Fig. 4f and g, the bending angle of HsGDY-M was consistently maintained over 250 cycles, illustrating its excellent cycling stability. Additionally, HsGDY-M is not only effective as a monolayer film with outstanding responsiveness but also enhances other materials by imparting stimulus-responsive capabilities to those lacking such properties. For instance, as shown in Fig. 4h, HsGDY can be directly grown on copper foil [34]. Placing this assembly in an acetone atmosphere, as depicted in Fig. 4i, allows for the curvature of the copper foil to be altered in response to changes in acetone concentration. This transfer of stimulus-response characteristics broadens the potential applications of HsGDY. These methods for growing graphdiyne on various substrates could lead to even more innovative applications in the future [35].

**Figure 5.**
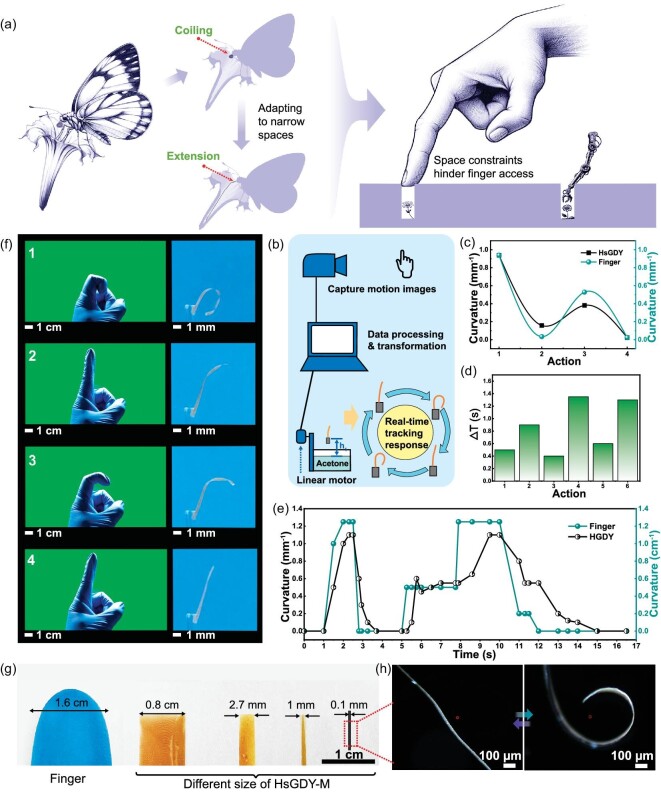
(a) Inspired by the adaptive stretching of a butterfly's proboscis in narrow flower stalks, this diagram illustrates a conceptual design for a micro-driving material suited for operation in narrow gaps. (b) The schematic diagram of the real-time tracking system shows its components and control principles. (c) The curvature of HsGDY corresponds with different finger movements. (d) The difference in response time between fingers and HsGDY under different actions. (e) The curvature changes of both the finger and HsGDY during tracking. (f) Photos comparing the positions of human fingers and HsGDY during the tracking process, visually demonstrating the system's ability to mirror human movements. (g) Photos displaying fingers and HsGDY-M of different sizes, providing a visual comparison of their dimensions. (h) Optical microscopy images reveal the reversible and controllable deformation of HsGDY-M, with a width of 100 μm.

Another interesting point of inspiration drawn from the butterfly proboscis is its ability to expand and contract, which enables butterflies to access nectar through narrow gaps, despite their large bodies. Small spaces often limit the effective manipulation of human fingers, as shown in Fig. 5a. A system has been set up here to achieve miniaturization of HsGDY-M to replicate the movement of human fingers and convert human finger movements into HsGDY-M movements. As shown in Video 3, HsGDY-M demonstrates an impressive ability to simulate human finger movements. When the finger is bent, the HsGDY-M on the right side will also bend similarly, and when the finger is unfolded, the HsGDY-M will also unfold. It is worth noting that HsGDY-M can maintain this semi-curved state when the fingers are held in a semi-curved position. This behavior conforms to the continuous and controllable deformation characteristics of HsGDY-M. Figure 5b shows various motion scenarios and the corresponding responses of HsGDY-M in tracking finger movements. The fundamental design principle of the system is depicted in Fig. 5c. Industrial cameras record motion images of fingers, and the computer applies a polynomial fitting to detect the edges of fingers, subsequently calculating the curvature at the midpoint of the polynomial curve. Next, based on these curvature readings, the system computes the precise position the motor should move to. The system determines the position, which presets the liquid level distances corresponding to various curvatures of HsGDY-M. By adjusting the height of the container holding the liquid via a linear motor to align with these distances, HsGDY-M is able to accurately replicate finger movements, as shown in Fig. S14. As shown in Fig. 5d, the bending angles of HsGDY-M vary depending on the finger's posture. During the motion tracking process, there is a lag time of ∼0.7 seconds before HsGDY-M deforms correspondingly, as shown in Fig. 5e. Furthermore, as illustrated in Fig. 5f, HsGDY-M reliably replicates curvature through real-time response, despite experiencing some delays in its reaction to changes in curvature. The ability of HsGDY-M to flexibly track finger movements underscores its potential as a microrobotic arm in various operational contexts. This capability of HsGDY-M, which combines accurate curvature replication with prompt response, positions it as a promising candidate for advanced applications in microrobotics. Furthermore, miniaturizing actuation materials is crucial for enhancing the operational range and enabling complex maneuvers in confined spaces. As shown in Fig. 5g, various sizes of HsGDY are compared with finger sizes, demonstrating that HsGDY can be tailored to specific dimensions according to specific requirements. Notably, HsGDY exhibits excellent controllable response characteristics at a size of 100 μm, as illustrated in Fig. 5h. This indicates its potential for application in micro-devices. In conclusion, HsGDY's versatility and controllable response at micro sizes make it a promising material for advanced technological applications.

## CONCLUSION

In conclusion, the development of the HsGDY-M artificial muscle, drawing inspiration from the morphological features of the butterfly's proboscis, epitomizes a transformative advance in actuation technology. Engineered from a HsGDY film with an innovative asymmetrical structure, this material not only facilitates rapid and reversible deformations but also enables precise dimensional tuning from ∼1 cm down to 100 μm. HsGDY-M operates robustly across environmental conditions, maintaining functional integrity even at temperatures as low as −25°C. Its beneficial responsiveness is transferable, enabling the transformation of ‘inert’ objects like copper foil into actuators via surface bonding. Its responsiveness and versatility are further demonstrated through its integration into robotic systems, where it significantly enhances load-bearing capacities and dynamic positioning capabilities. Moreover, its application in real-time movement-tracking systems demonstrates its potential to revolutionize precision in microscale robotic applications and biomedical devices. These materials operate accurately and reliably even under harsh environmental conditions, enabling precise control of microscale movements and marking a significant advance in the field of artificial muscles.

## Supplementary Material

nwae400_Supplemental_File
