# Supplementary material for: Bio-inspired carbon-based artificial muscle with precise and continuous morphing capabilities
Source: Natl Sci Rev. 2024 Nov 8;12(1):nwae400. doi: 10.1093/nsr/nwae400 (PMC11702649; doi:10.1093/nsr/nwae400)
Supplement: nwae400_Supplemental_File [file nwae400_supplemental_file.zip › Teaser text.docx]

Inspired by a butterfly’s proboscis, this carbon-based artificial muscle offers precision, rapid morphing and adaptability, heralding potential breakthroughs in robotics and enabling real-time tracking for medical applications.
